# PROMATER: protocol for a randomized controlled trial of a digital self-guided intervention to promote perinatal mental health and reduce self-criticism

**DOI:** 10.3389/fpsyt.2026.1943486

**Published:** 2026-09-17

**Authors:** Bruna Cardoso Gerhardt, Maria Eduarda dos Santos, Valentino Bohn Debastiani, Elizabeth Werner, Catherine Monk, Adriane Xavier Arteche

**Affiliations:** 1 School of Health and Life Sciences, Pontifical Catholic University of Rio Grande do Sul, Porto Alegre, Brazil; 2 Division of Women’s Mental Health, Department of Obstetrics and Gynecology, Columbia University Irving Medical Center, New York, NY, United States

**Keywords:** perinatal mental health, self-criticism, self-compassion, online intervention, postpartum depression, anxiety

## Abstract

**Background:**

Perinatal mental health represents a significant public health concern given the high prevalence of mental disorders during pregnancy and the postpartum period and their impact on maternal well-being, mother-infant bonding, and child development. Emerging evidence identifies self-criticism as a transdiagnostic vulnerability factor associated with psychological distress in this period, while self-compassion has been recognized as an important protective factor. In Brazil, access to preventive perinatal mental health interventions remains limited. This paper describes the protocol of a randomized controlled trial (RCT) evaluating PROMATER – an online program designed to promote perinatal mental health by targeting self-criticism and self-compassion.

**Methods:**

A total of 130 pregnant women between 28 and 34 weeks of gestation will enroll in a two-arm RCT with a dismantling design. Participants will be randomized (1:1 ratio) to either the PROMATER intervention or to an active control group. Participants and data analysts will be blinded to group allocation. PROMATER consists of a 3-week program (6 modules) addressing maternal expectations, self-criticism, perinatal mental health, social support, self-compassion, and mindfulness and mother-infant bonding. The active control offers a structurally equivalent program covering the same topics, except for the modules specifically targeting these transdiagnostic processes. Primary outcomes are self-compassion, assessed via the compassionate self-responding (CSR) score of the Self-Compassion Scale, and self-criticism, operationalized through the uncompassionate self-responding (USR) score. Secondary outcomes include postpartum depression, anxiety, quality of life, social support, and mother-infant bonding. Assessments occur at baseline (T1), post-intervention (T2), and 3-month postpartum follow-up (T3). Data will be analyzed using Linear Mixed Models (LMM) following the Intention-to-Treat (ITT) principle.

**Discussion:**

Evaluating the effectiveness of a brief, accessible, online intervention has the potential to address the need for an evidence-based approach designed to promote maternal well-being during pregnancy and the postpartum period. The intervention is designed to maximize reach and minimize cost, with the potential to scale up and incorporate in routine perinatal care. The active control condition will allow examination of whether directly targeting self-criticism and self-compassion confers incremental benefit over structured perinatal psychoeducation alone.

**Systematic review registration:**

https://ensaiosclinicos.gov.br/, identifier RBR-99tjfz5.

## 1 Introduction

Mental health during pregnancy and the first year postpartum is a critical determinant of both maternal and child well-being. Perinatal mood and anxiety disorders (PMADs) affect approximately one in four women in low- and middle-income countries (LMICs) (1, 2) and are associated with substantial impairments in maternal functioning, quality of life, and overall health (3). Beyond their impact on mothers, PMADs are linked to adverse cognitive, emotional, and socioemotional outcomes in offspring, with effects that may extend into childhood and adulthood (4–6). Consequently, when left untreated, these conditions impose considerable social and economic burdens on individuals, families, and healthcare systems (7).

Despite the availability of effective preventive interventions, access to perinatal mental health care remains severely limited across LMICs (8). Even in upper-middle-income countries such as Brazil, structural, geographic, and socioeconomic barriers within the public health system leave many women without adequate support during pregnancy and the postpartum period (9). This treatment gap highlights the urgent need for scalable and accessible preventive approaches capable of reaching women before symptoms become clinically significant.

Current healthcare systems continue to prioritize the treatment of established disorders rather than the promotion of psychological well-being and resilience earlier in the perinatal period. This reactive model is particularly concerning given that postpartum depression frequently originates during pregnancy (10). Early identification of vulnerability factors and timely preventive support are therefore essential for reducing the risk of adverse outcomes, including maternal morbidity and mortality (11), preterm birth (12, 13), impaired infant neurodevelopment (4, 14), and disruptions in mother-infant bonding (15).

Reflecting growing recognition of perinatal mental health as a public health priority, Brazil’s Law No. 14,721 (2023) expanded the statutory right to psychological support within the Unified Health System (SUS). However, translating this policy commitment into accessible and scalable care remains challenging. Given that more than 90% of Brazilian households have internet access (16), digital interventions represent a promising strategy for overcoming geographical and financial barriers. Digital approaches may be particularly well suited for preventive care because they can be delivered early, at relatively low cost, and with minimal demands on already overburdened healthcare systems. Consistent with this potential, recent meta-analyses indicate that digital prenatal interventions can significantly reduce depressive and anxious symptoms (17, 18).

The transition to motherhood involves substantial psychological and relational reorganization, during which women navigate shifting identities, new caregiving demands, and heightened emotional vulnerability (19). This period is also shaped by cultural expectations and idealized representations of motherhood that influence women’s perceptions of maternal competence and adequacy. When lived experience diverges from these expectations, women may engage in harsh self-evaluation, attributing perceived failures to personal inadequacy rather than to the inherent challenges of the perinatal transition (11, 20). These self-evaluative responses represent both a psychological risk factor and a potential target for preventive intervention.

Understanding these self-evaluative processes requires a transdiagnostic lens. Recent developments in clinical psychology emphasize the importance of psychological processes that cut across diagnostic categories (21–23), offering a flexible framework for addressing the complex, frequently comorbid presentations characteristic of perinatal mental health difficulties (11). Among these processes, self-criticism – defined as a harsh and punitive self-evaluative stance in response to perceived failures or inadequacies (24) – has emerged as a particularly relevant transdiagnostic vulnerability factor. High levels of self-criticism are associated with a broad range of psychological difficulties, including depression and anxiety (25, 26), as well as poorer therapeutic outcomes and reduced engagement in self-care practices (27).

Evidence from studies using heart rate variability and functional neuroimaging indicates that self-criticism is strongly associated with activation of the threat system (28, 29). In contexts characterized by high demands and social comparison – such as the perinatal period – women may adopt self-evaluative patterns marked by self-reproach and fear of negative evaluation (20). These patterns can trigger physiological stress responses, including activation of the hypothalamic–pituitary–adrenal (HPA) axis, increased cortisol levels, and heightened autonomic reactivity (30). Dysregulation of these systems during the perinatal period may adversely affect both maternal health and infant development through mechanisms such as transplacental cortisol exposure and reduced maternal emotional responsiveness postpartum (32). Taken together, these findings indicate that elevated self-criticism may contribute to stress-related physiological cascades with meaningful consequences for maternal and infant outcomes.

As a conceptual and therapeutic counterpart to self-criticism, self-compassion – defined as the capacity to respond to personal suffering with kindness, understanding, and recognition of shared human experience – is associated with greater emotional resilience and psychological well-being (24, 31). Interventions designed to cultivate self-compassion, such as Compassion-Focused Therapy (33) and the Mindful Self-Compassion program (34), have shown promising results across diverse populations. In the perinatal context, compassion-based interventions have demonstrated potential for improving maternal mental health outcomes, including increases in self-compassion and reductions in depressive symptoms (35–37). However, many existing interventions focus primarily on promoting self-compassion without directly addressing entrenched patterns of self-criticism. This limitation may reduce their effectiveness, particularly among individuals who experience fears of compassion – psychological barriers that inhibit the capacity to respond to oneself with kindness (38).

The intrapersonal mechanisms described above – self-criticism and self-compassion – do not operate in isolation. Perinatal psychological vulnerability is embedded in a relational and contextual matrix in which social support plays a central moderating role. Perceived social support from partners, family, and peers buffers against the onset and severity of perinatal depressive and anxious symptoms and sustains gains over time (11, 39). Qualitative evidence highlights the breadth of support that pregnant women value – encompassing emotional support from female networks, practical and financial assistance, and access to health information – with gaps in any of these domains associated with increased stress, loneliness, and poorer maternal outcomes (40). Critically, social support also modulates the self-evaluative processes described above: women with stronger support networks report lower levels of self-criticism and shame (30), suggesting that interpersonal and intrapersonal mechanisms are mutually reinforcing targets for preventive intervention.

A further mechanism through which these processes can be addressed is mindfulness. Mindfulness-based approaches have demonstrated efficacy in reducing perinatal anxiety and depressive symptoms, in part by fostering present-moment awareness and reducing ruminative and self-critical thinking (41, 42) - directly attenuating one of the core processes identified above. Beyond its effects on self-criticism, mindfulness practice supports the development of the mother-infant relationship by cultivating non-judgmental present-moment attention, creating conditions for greater maternal emotional availability and sensitivity toward the infant (43). Mother–infant bonding, defined as the affective relationship that develops from mother to infant during the perinatal period, is both an outcome of maternal mental health and a protective factor for infant cognitive and socioemotional development (15). Interventions that explicitly address bonding within a preventive framework may therefore help interrupt the intergenerational transmission of psychological risk, making it a clinically meaningful target even before birth (44).

Several digital and multicomponent programs have been developed to prevent perinatal depression and anxiety, yet none explicitly targets self-criticism as a primary therapeutic mechanism. Among the landmark counseling programs recommended by the U.S. Preventive Services Task Force, ROSE, an interpersonal therapy-based group intervention addressing social support and role transitions (45, 46), and Mothers and Babies, a cognitive-behavioral program targeting mood management and activity engagement (47), have demonstrated robust efficacy, but are delivered in person and do not address self-criticism or self-compassion. In the digital domain, MomMoodBooster (48) and mobile mindfulness-based interventions (42) have reduced perinatal symptoms but focus on mood regulation and present-moment awareness rather than compassion-based processes. The most structurally comparable program, Be a Mom (35), integrates self-regulatory skills, psychoeducation, and emotional regulation, yet does not operationalize self-compassion or self-criticism as distinct targets. Consistent with this pattern, a recent meta-analysis of digital interventions reported moderate overall effects but substantial heterogeneity in components and limited mechanistic specificity (18).

Taken together, this evidence identifies a set of interrelated and modifiable mechanisms as targets for perinatal prevention. Although multicomponent digital programs have begun to combine several of these targets within a single intervention (18, 35), none has operationalized self-criticism as a primary therapeutic target or used a dismantling design to test its incremental contribution over an active psychoeducational comparator - particularly in LMICs or non-English-speaking contexts. PROMATER (Program to Promote Perinatal Mental Health), a digital self-guided intervention, was designed to address precisely this gap. The present paper reports the protocol of a randomized controlled trial (RCT) evaluating its efficacy.

Recognizing the common challenge of participant retention in digital health interventions (49, 50), the program incorporates evidence-based engagement strategies. It is hosted on a responsive platform accessible across multiple devices, featuring short psychoeducational videos (under five minutes) designed to accommodate the time constraints and fatigue characteristic of the perinatal period. Adherence is further supported through multimodal content, including guided audio practices, visually engaging infographics, and interactive quizzes, supplemented by automated email and text message reminders.

To examine the specific contribution of the self-criticism and self-compassion components, the study employs an active control condition. Participants in the control group receive a structurally equivalent intervention – covering expectations of motherhood, psychoeducation on perinatal disorders, social support, mindfulness, and mother-infant bonding – but without the modules specifically targeting self-compassion and self-criticism. This dismantling design enables examination of whether directly addressing these transdiagnostic mechanisms confers incremental benefit over standard preventive psychoeducation. To our knowledge, this is one of the first dismantling RCTs of a digital compassion-based intervention conducted within a perinatal population in Brazil, thereby contributing novel evidence on the active ingredients of digital perinatal mental health programs.

## 2 Objectives

### 2.1 Primary objective

To evaluate the effectiveness of the PROMATER intervention in enhancing self-compassion, assessed via the compassionate self-responding (CSR) score, and reducing self-criticism, operationalized through the uncompassionate self-responding (USR) score, of the Self-Compassion Scale (SCS) among perinatal women. We hypothesize that participants in the PROMATER group will demonstrate significantly greater increases in CSR and greater reductions in USR compared to the active control group.

### 2.2 Secondary objectives

To evaluate the effects of PROMATER on secondary outcomes: postpartum depression, anxiety, quality of life, perceived social support and mother-infant bonding. We hypothesize that participants in the PROMATER group will demonstrate significantly greater reductions in depressive and anxious symptoms compared to the active control group, and greater improvements in quality of life, social support and quality of mother-infant bonding.

## 3 Methods and analysis

### 3.1 Trial design

This study employs a two-arm randomized controlled trial design comparing the PROMATER intervention with an active control condition, with participants allocated in a 1:1 ratio. Participants and data analysts will be blinded to group allocation, whereas investigators responsible for intervention management and participant monitoring will not remain blinded following randomization.

PROMATER is conceptualized as a selective and indicated preventive intervention, consistent with Gordon’s (51) prevention taxonomy. The trial targets pregnant women in the third trimester, a population at elevated risk for postpartum depression and anxiety, with the primary aim of reducing the onset or severity of postpartum symptoms. Women with current gestational depressive or anxiety symptoms are not excluded, as the intervention targets postpartum rather than gestational psychopathology; PROMATER is not designed or claimed to treat current clinical conditions.

### 3.2 Participants

Participants will be 130 Brazilian pregnant women recruited online across Brazil.

### 3.3 Eligibility criteria

#### 3.3.1 Inclusion criteria

1. Women aged 18 years or older
2. Pregnant women in the third trimester (>28 weeks and <34 weeks of gestation)
3. Ability to read and understand Portuguese
4. Access to internet and digital device

The 28–34-week gestational window was selected to deliver the intervention during the third trimester, when anxiety symptoms tend to increase, while maintaining sufficient time for participants to complete the program before the expected delivery. This timing was refined following pilot testing and expert review, which highlighted the importance of delivering preventive content sufficiently close to the postpartum period to maximize its relevance.

#### 3.3.2 Exclusion criteria

##### 3.3.2.1 Self-reported severe psychiatric conditions requiring immediate care

Mental health history is assessed through participant self-report, including previous professionally diagnosed mental health conditions, current psychological or psychiatric treatment, and current use of psychiatric medication. Current depressive and anxiety symptoms are additionally assessed using the EPDS and GAD-7, respectively, with EPDS Item 10 specifically monitored as part of the study’s safety procedures. A previous psychiatric diagnosis, elevated depressive or anxiety symptoms, current treatment, or psychotropic medication use does not automatically result in exclusion. The exclusion criterion is intended to identify participants presenting a current psychiatric condition requiring immediate or intensive clinical care that would make participation in a low-intensity, self-guided preventive intervention inappropriate. PROMATER is not intended to substitute for treatment of established psychiatric disorders.

### 3.4 Interventions

#### 3.4.1 The PROMATER program (experimental condition)

The PROMATER intervention is a digital, self-guided program developed to target transdiagnostic processes associated with perinatal mental health. The program is delivered via a responsive, mobile-friendly web platform, structured into weekly modules. To enhance user engagement and mitigate the high attrition rates typically observed in digital health (49, 50), the intervention incorporates a multi-modal microlearning design. Each module consists of short psychoeducational videos (around 5 minutes), accompanied by guided audio practices, and visually engaging infographics summarizing key concepts. Interactive quizzes are strategically placed to reinforce knowledge retention. To promote adherence, participants receive automated reminders via email or text message throughout the intervention period.

The theoretical framework of PROMATER integrates elements from Compassion-Focused Therapy (CFT), Mindful Self-Compassion (MSC), and Process-Based Psychology. The intervention was developed following an extensive review of the literature on theoretical models and digital programs aimed at promoting perinatal mental health. To ensure alignment with clinical practice and the needs of pregnant women, two specialist psychologists with experience in perinatal mental health reviewed the protocol and suggested modifications to improve the intervention. In addition, two women from the target population were given access to PROMATER prior to data collection to evaluate aspects such as clarity of content, usability, practical applicability, and potential for engagement.

To enhance linguistic and cultural appropriateness for the Brazilian context, the intervention was developed using simple and accessible language, avoiding unnecessary technical terminology. CFT- and MSC-informed exercises were adapted into Portuguese using accessible wording, and all video content includes optional Portuguese subtitles. Visual materials were selected to reflect the diversity of Brazilian women and families, including diversity in racial and ethnic backgrounds, physical characteristics, and family configurations.

The program was initially designed to be delivered over six weeks, based on comparable interventions. Following pilot testing and expert review, the duration was shortened to three weeks to reduce participant burden and potential attrition. As video duration was also reduced from approximately 15 to 5 minutes, two modules are released per week to maintain adequate exposure to intervention content while preserving a brief and flexible format. The intervention structure is provided in **Additional File 1**, with a detailed description of each module, including content and key exercises.

#### 3.4.2 Control condition

The active control group receives a structurally equivalent digital program delivered through the same platform, with the same visual interface, delivery format, and module structure as PROMATER. This design ensures that both groups have equivalent module exposure, platform access, and engagement format, thereby controlling for non-specific effects of digital participation such as attention, expectancy, and psychoeducation.

Both conditions share four modules with identical content: Module 1 (Expectations and Idealization of Motherhood), Module 3 (Perinatal Mental Health), Module 4 (Social Support), and Module 6 (Mindfulness and Mother-Infant Bonding). The active control condition differs from PROMATER exclusively in Modules 2 and 5, which in the control arm are replaced by psychoeducational content on perinatal health topics unrelated to self-criticism and self-compassion. This substitution ensures dosage equivalence in module structure – each control module contains a psychoeducational video, infographic, and interactive quiz – while isolating the theoretical components under investigation.

##### 3.4.2.1 Module 2 (control) – fetal development in the third trimester

This module provides accessible psychoeducation on fetal development between 28 and 40 weeks of gestation. Content covers the maturation of key physiological systems (neurological, pulmonary, and sensory), the development of fetal sensory capacities including auditory responsiveness to the mother’s voice, and key developmental milestones across the third trimester. This content was selected for its direct relevance to the perinatal period and its potential to foster early relational awareness of the infant, while remaining theoretically inert with respect to the active components of self-criticism and self-compassion.

##### 3.4.2.2 Module 5 (control) – preparing for birth

This module offers practical and informational content on birth preparation. Topics include the physiological stages of labor, distinguishing signs of labor onset, types of delivery and their respective indications, and preparation of the hospital bag. The module aims to provide information to support women’s sense of preparedness and agency ahead of delivery, without introducing any compassion-based or self-evaluative content.

### 3.5 Outcomes

#### 3.5.1 Sociodemographic and clinical questionnaire

A custom-designed questionnaire will be used to characterize the sample and explore potential moderating variables. It includes demographic and clinical data such as age, marital status, self-declared race/ethnicity, sexual orientation, occupational status, education, monthly household income, parity, history of mental disorders, and self-perceived social support. (Administered at T1).

#### 3.5.2 Obstetric and postpartum experience questionnaire

This 19-item investigator-developed questionnaire is administered at T3 to descriptively characterize participants’ obstetric, birth, and early postpartum experiences. It assesses gestational age at delivery, type of birth, medical interventions and complications, maternal and neonatal hospitalization, breastfeeding, infant medication use, and perceived infant temperament. Items use multiple-choice, multiple-response, Likert-type, and open-ended response formats. Items will be analyzed descriptively, and no total or subscale scores will be calculated. The questionnaire is not an outcome measure and will not be used to assess intervention effectiveness. The complete questionnaire is provided in the Supplementary Material. (Administered at T3).

#### 3.5.3 Primary outcomes

##### 3.5.3.1 Self-compassion and self-criticism

Self-Compassion Scale (SCS; 24; Brazilian version by 52): The SCS consists of 26 items rated on a 5-point Likert scale. Consistent with previous research recommending separate examination of the positive and negative components of the SCS (53–56), the present study will calculate separate scores for compassionate self-responding (CSR; self-kindness, common humanity, and mindfulness) and uncompassionate self-responding (USR; self-judgment, isolation, and over-identification). This approach avoids potential interpretative limitations associated with combining positive and reverse-scored negative components into a single total score and allows examination of their potentially distinct intervention effects. In a previous Brazilian perinatal sample, both scores demonstrated good internal consistency (ω=0.92 and ω=0.91, respectively; 57). Internal consistency will also be estimated in the present sample. (Administered at T1, T2, and T3).

#### 3.5.4 Secondary outcomes

##### 3.5.4.1 Perinatal depression

Edinburgh Postnatal Depression Scale (EPDS; 58; Brazilian version by 59): A 10-item self-report scale designed to screen for depressive symptoms in the past seven days. The Brazilian version suggests a cutoff score of ≥10 as indicative of clinically significant symptoms. It demonstrates good internal consistency (α=0.87). (Administered at T1, T2, and T3).

##### 3.5.4.2 Perinatal anxiety

Generalized Anxiety Disorder-7 (GAD-7; 60; Brazilian version by 61): A 7-item instrument assessing anxiety symptoms over the past two weeks. Scores range from 0 to 21, classified as minimal (0–4), mild (5–9), moderate (10–14), or severe (15–21). The Brazilian version shows high reliability (α=0.91). (Administered at T1, T2 and T3).

Postpartum Specific Anxiety Scale – Research Short Form – Global Crises (PSAS-BR-RSF-C; 62; Brazilian version by 63): A 12-item scale tailored to the postpartum period, measuring maternal competence, infant safety, and psychosocial adjustment. A cutoff of 26 indicates high postpartum anxiety. The Brazilian validation reported a reliability of ω=0.77. (Administered at T3).

##### 3.5.4.3 Mother-infant bonding

Postpartum Bonding Questionnaire (PBQ; 64; Brazilian version by 65): A 12-item scale evaluating difficulties in the maternal-infant relationship. Higher scores indicate greater bonding impairment. The validated Brazilian version assesses three factors: Loss of Interest, Rejection and Anger, and Anxiety/Risk of Abuse, with a general “Impaired Bonding” factor (ω=0.83). (Administered at T3).

##### 3.5.4.4 Quality of life

World Health Organization Quality of Life – Bref (WHOQOL-BREF; 66; Brazilian version by 67): A 26-item instrument measuring perceived quality of life across four domains: physical, psychological, social relationships, and environment. Higher scores reflect better quality of life. Internal consistency for the Brazilian version ranges from α=0.65 to 0.80. (Administered at T1, T2, and T3).

##### 3.5.4.5 Social support

Medical Outcomes Study Social Support Survey (MOS-SSS; 68; Brazilian version by 69): A 19-item scale evaluating functional social support across domains such as emotional, material, and affective support. Higher scores indicate greater perceived support. The Brazilian version shows excellent reliability (α=0.74 to 0.95). (Administered at T1, T2, and T3).

### 3.6 Participant timeline

The schedule of enrollment, interventions, and assessments for all participants is presented in **Table 1**, following the SPIRIT 2025 recommended format.

**Table 1:** Participant timeline: schedule of enrollment, interventions, and assessments.

	Study procedure			
Timepoint	*t* *_1_*	Intervention start	*t* *_2_*	*t* *_3_*
	>28 weeks <34 weeks gestation	Week 0	Week 3	3 months postpartum
Enrollment				
Eligibility screen	X			
Informed consent	X			
Randomization	X			
Intervention/comparator				
PROMATER		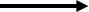		
Active Control		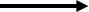		
Assessments				
Sociodemographic Questionnaire	X			
Primary outcomes				
SCS (Self-compassion subscale)	X		X	X
SCS (Self-criticism subscale)	X		X	X
Secondary outcomes				
EPDS	X		X	X
GAD-7	X		X	X
PSAS-BR-RSF-C				X
WHOQOL-BREF	X		X	X
MOS-SSS	X		X	X
PBQ				X
Obstetric and PP Questionnaire				X

SCS, Self-Compassion Scale; EPDS, Edinburgh Postnatal Depression Scale; GAD-7, Generalized Anxiety Disorder-7; PSAS-BR-RSF-C, Brazilian Portuguese version of the Postpartum Specific Anxiety Scale – Research Short-Form for use in global Crises; WHOQOL-BREF, World Health Organization Quality of Life–BREF; MOS-SSS, Medical Outcomes Study Social Support Survey; PBQ, Postpartum Bonding Questionnaire; Obstetric and PP Questionnaire, obstetric and postpartum questionnaire developed for this study.

If a participant gives birth before completing the intervention or before the scheduled T2 assessment, the antenatal T2 assessment will not be administered. Intervention exposure up to delivery will be documented, and the participant will remain eligible for the T3 postpartum follow-up.

### 3.7 Sample size

The sample size was estimated using G*Power software (version 3.1.9.7) based on a repeated-measures analysis of variance testing the within-between interaction, used as an approximation for the planned longitudinal group-by-time analysis. The calculation assumed a medium effect size (Cohen’s f = 0.25), informed by previous meta-analytic evidence indicating moderate effects of internet-delivered psychological interventions in perinatal populations and of self-compassion-related interventions on self-criticism (70, 71). Additional assumptions included two groups, three measurement occasions, a correlation of 0.50 among repeated measures, a nonsphericity correction of ϵ = 1.0, a two-sided alpha level of 0.05, and 95% statistical power. Under these assumptions, the minimum required sample size was 108 participants (54 per group). The recruitment target was prospectively increased by 20% to 130 participants (65 per group) to mitigate anticipated loss to follow-up. However, attrition in comparable self-guided perinatal digital interventions has sometimes exceeded this allowance. Accordingly, loss to follow-up and intervention adherence will be monitored and reported separately, and their implications for statistical power will be considered when interpreting the trial findings.

### 3.8 Recruitment

Participants will be recruited through a multi-channel strategy designed to enhance reach and sample diversity. Recruitment efforts will include organic and paid promotions on social media platforms such as Instagram and Facebook, using visually engaging posts and targeted advertisements aimed at women within specific gestational periods. Paid advertisements will also prioritize recruitment in low-income areas to increase the representativeness of the sample. In addition, digital flyers and project presentations will be distributed to healthcare professionals working within the maternal-child care network of the Brazilian Unified Health System (SUS) to facilitate access to women from diverse sociodemographic backgrounds. Paid social media advertisements are funded directly by the doctoral researcher.

### 3.9 Randomization

Before recruitment, a random allocation sequence was generated using the *random.org* List Randomizer. A list containing 65 assignments to each condition was randomized, generating a sequence of 130 allocations with a fixed 1:1 ratio. Following completion of the baseline assessment (T1), participants are sequentially assigned to the next available allocation in the predefined sequence.

The complete allocation sequence is concealed from personnel involved in recruitment and eligibility screening but is accessible to the independent researcher responsible for executing the allocation. This procedure ensures that recruitment personnel cannot influence or anticipate group assignments. Participants and data analysts remain blinded to treatment allocation throughout the trial.

### 3.10 Implementation

The enrollment process will be conducted by the primary research team, who will screen for eligibility and obtain informed consent. Following the completion of the baseline assessment (T1), the independent researcher will execute the randomization and assign the participant to the respective study arm. This procedure ensures that the personnel responsible for recruiting and assessing participants cannot influence the allocation sequence, thereby minimizing selection bias.

### 3.11 Blinding

Participants will remain blinded to group allocation and to the study’s primary hypothesis throughout the trial. Both study arms receive a structured digital intervention through the same platform, with an identical visual interface and delivery format, and participants will not be informed which condition contains the components specifically targeting self-criticism and self-compassion. This approach is intended to minimize expectancy and performance-related biases associated with knowledge of treatment allocation.

Investigators responsible for intervention management and participant monitoring will not remain blinded following randomization, as knowledge of group allocation is required for intervention delivery and monitoring. Data analysts conducting the primary statistical analyses will remain blinded to allocation and will receive datasets in which study groups are identified using masked group codes. Group identities will be revealed only after the primary analyses have been completed.

### 3.12 Data collection methods

Data collection will be conducted exclusively through online self-report questionnaires. To ensure high-quality data and participant privacy, the following procedures will be implemented. All assessments will be administered via the Qualtrics platform, which provides an encrypted and secure environment for clinical data collection. The digital platform will employ automated checks for missing data (e.g., “force response”), preventing participants from progressing to subsequent modules if critical items are left unanswered. Also, intervention engagement and exposure will be monitored using data automatically recorded by the Hotmart platform, including overall program progress, the number and proportion of completed content items, completion of individual modules, and access frequency and dates. Module completion will be reported separately and will not, by itself, be interpreted as evidence of meaningful adherence to the intervention.

Baseline variables are assigned prespecified analytic roles as follows. Depressive symptoms at baseline (EPDS T1), parity (primiparous vs. multiparous), concomitant psychological or pharmacological treatment at baseline, and gestational age at enrollment are designated as prespecified covariates in all primary and key secondary analyses. Obstetric history and partner/relationship support are collected for descriptive purposes and will be reported as part of the sample characterization. These variables may additionally be explored as potential moderators in secondary analyses, which are declared explicitly as exploratory.

Information on current psychological and pharmacological treatment is collected at baseline (T1). Although treatment status was not reassessed at T2, this variable will be added to the T3 assessment to document any changes during the follow-up period and will be reported descriptively alongside baseline treatment status.

### 3.13 Data management

The management of data will strictly adhere to the Brazilian General Data Protection Law (LGPD) and international ethical standards for clinical trials. All raw data will be stored on password-protected servers with limited access, hosted by the survey platform and the research institution. To protect participant identity, data will be anonymized immediately after the initial data cleaning process. Personal identifiers (e.g., names, emails) will be replaced by a unique participant ID code and stored in a separate, encrypted master file. The final dataset will be accessible only to authorized researchers directly involved in the statistical analysis. Data will be kept for a minimum of five years following the publication of the results, after which it will be permanently deleted in accordance with institutional ethical guidelines.

### 3.14 Statistical analysis

The analyses will be conducted using R in R Studio. Participant characteristics at baseline will be compared between groups using independent samples t-tests for continuous variables and Chi-square (χ2) tests for categorical variables. Any observed imbalances will be reported transparently and explored in sensitivity analyses only.

The primary analysis will follow the intention-to-treat (ITT) principle, including all randomized participants regardless of module completion status. The primary statistical approach will use Linear Mixed Models (LMM) with group (PROMATER vs. active control), time (T1, T2, T3), and the group × time interaction as fixed effects, and participant as a random intercept. Time will be treated as a categorical variable with T1 as the reference category, given the unequal and theoretically meaningful intervals between assessment points. The primary effect of interest is the group × time interaction, which tests whether the trajectory of change in CSR and USR differs between arms. The primary contrasts of interest are T1→T2 and T1→T3, testing immediate post-intervention and follow-up effects respectively. Missing data will be handled under the missing-at-random (MAR) assumption, as LMM uses all available data without requiring complete cases or imputation. In the LMM, T1, T2, and T3 are included as the repeated outcome measurements defining the longitudinal trajectory.

Two primary outcomes (CSR and USR) will be tested with a Bonferroni-corrected significance threshold of α = .025 each. Although the sample size was calculated at α = .05, the Bonferroni correction at α = .025 was subsequently specified to control the familywise error rate across the two primary outcomes. The trial is therefore likely to be underpowered for the multiplicity-adjusted analyses, and any impact on statistical power will be reported transparently in the primary outcomes paper.

Postpartum depression (EPDS) and anxiety (GAD-7) are designated as key secondary outcomes and will be analyzed in a hierarchical testing procedure, conditional on significance of at least one primary outcome at α = .025. Results for key secondary outcomes will be reported regardless of primary outcome results, with interpretation adjusted accordingly. All remaining secondary outcomes (quality of life, mother-infant bonding, social support, postpartum-specific anxiety) will be treated as exploratory; no multiplicity correction will be applied, and findings will be reported with effect sizes and 95% confidence intervals to support clinical interpretation.

For outcomes assessed exclusively at T3 - specifically postpartum anxiety (PSAS) and mother-infant bonding (PBQ), which are conceptually meaningful only in the postpartum period - a separate analysis will be conducted using ANCOVA with group as a fixed factor. Because neither outcome has a direct baseline measurement in this design, proxy baseline covariates will be used. GAD-7 at T1 will serve as the baseline covariate for the PSAS analysis, as general anxiety symptoms at baseline represent the closest available measure of anxiety vulnerability. For the PBQ analysis, EPDS at T1 will be used because antenatal depressive symptoms are an established predictor of postpartum bonding difficulties.

The following covariates are prespecified for inclusion in all primary and key secondary analyses based on theoretical and empirical grounds: depressive symptoms at baseline (EPDS T1), parity (primiparous vs. multiparous), concomitant psychological or pharmacological treatment at baseline, and gestational age at enrollment. Obstetric history and partner/relationship support are collected for descriptive purposes and will be reported as part of the sample characterization. These variables may additionally be explored as potential moderators in secondary analyses, which are declared explicitly as exploratory.

### 3.15 Trial monitoring

Trial conduct will be monitored continuously by the research team through platform analytics, data quality checks, and participant feedback. Given the low-risk nature of the intervention, a formal Data Monitoring Committee will not be established. The research team will be responsible for monitoring data quality and participant safety.

## 4 Discussion

This protocol describes the rationale and methodology for a randomized controlled trial evaluating the effectiveness of PROMATER, a brief, self-guided digital intervention designed to promote perinatal mental health by targeting self-criticism and self-compassion. To our knowledge, this represents one of the first dismantling RCTs of a compassion-based digital intervention conducted within a perinatal population in an upper-middle-income country, contributing novel evidence on the active ingredients of digital perinatal mental health programs. The intervention is designed to optimize reach and minimize implementation costs, with potential for integration into routine perinatal care within the Brazilian public health system.

A major strength of this protocol is its focus on the third trimester of pregnancy (weeks 28–34), a period often characterized by heightened anxiety and increasing psychological vulnerability, and one that is particularly sensitive for preventive intervention given that postpartum depression frequently originates during pregnancy (10). By intervening before the onset of clinical symptoms, PROMATER aims to build psychological resources – particularly self-compassion and reduced self-criticism – that may buffer against the deterioration in mental health commonly observed across the perinatal transition. The three-assessment design (T1, T2, T3) further allows examination of both immediate post-intervention effects and the sustainability of gains at three months postpartum, a period of particularly high vulnerability for PMADs.

### 4.1 Interpretation of possible outcomes

In consideration of the full range of possible trial outcomes and their scientific implications, three scenarios are anticipated:

#### 4.1.1 Scenario 1 – PROMATER outperforms the active control on primary outcomes

If participants in the PROMATER arm demonstrate significantly greater improvements in self-compassion and reductions in self-criticism relative to the active control at T2 and T3, this would provide support for the theoretical model underlying the intervention - specifically, that directly targeting these transdiagnostic processes confers incremental benefit over general psychoeducation. Such a finding would strengthen the case for including self-compassion and self-criticism components as active ingredients in digital perinatal mental health programs and would support the broader transdiagnostic framework applied to perinatal populations (11). It would also have direct implications for the design of future scalable interventions, suggesting that compassion-focused content should be retained rather than subsumed within broader psychoeducational programs.

#### 4.1.2 Scenario 2 – both conditions improve equivalently (null result on between-group differences)

If both groups demonstrate within-group improvements with no significant between-group differences, this would suggest that the explicit self-compassion and self-criticism components did not confer measurable incremental benefit over the active comparator. This outcome would not invalidate the preventive value of the broader program; rather, it would redirect inquiry toward the shared components as potential active mechanisms of change, consistent with evidence that common therapeutic factors account for a substantial proportion of intervention effects across formats (11, 21). Importantly, within-group improvements would not establish the efficacy of either condition in the absence of a no-intervention comparison group, as observed changes could reflect non-specific effects, natural perinatal symptom trajectories, or regression to the mean. A null between-group result would nonetheless inform intervention development: if equivalent outcomes are achievable without explicit compassion-based training, a simplified program may represent a more scalable and cost-effective approach for population-level prevention.

#### 4.1.3 Scenario 3 – neither condition produces significant improvement

If neither arm demonstrates significant pre-to-post improvements, this would raise important questions about the adequacy of a three-week self-guided digital format for producing measurable psychological change in a perinatal population. This outcome would not necessarily invalidate the theoretical rationale for targeting self-compassion and self-criticism but would suggest that brief self-guided delivery may be insufficient without additional clinical support, greater intervention intensity, or different engagement mechanisms. Such findings would contribute to the growing literature on the boundary conditions of digital mental health interventions and would inform recommendations for future program development – including the potential value of blended delivery models combining digital content with human support.

## 5 Limitations

Several limitations of the current protocol are acknowledged. First, the self-guided nature of the intervention precludes verification of meaningful engagement with therapeutic content beyond module completion metrics. Participants who complete all modules may vary substantially in the depth of their engagement with the psychological exercises, which could attenuate observed effects. Second, despite efforts to equalize module structure across conditions, the self-criticism and self-compassion modules in PROMATER contain a higher volume of video content reflecting their theoretical centrality to the intervention. This residual dosage difference represents an inherent limitation of the dismantling design and will be monitored through differential attrition analysis between arms and reported transparently in the results. Third, recruitment primarily through social media may limit sample representativeness and introduce self-selection bias, as women who engage with online mental health research may differ from the broader population of pregnant women. Online recruitment may also underrepresent women with limited internet access or lower digital literacy, potentially limiting the generalizability of the findings. Fourth, the follow-up period is limited to three months postpartum; therefore, the study will not determine whether intervention effects persist across the broader postpartum period. Finally, the exclusive reliance on self-report measures is a limitation shared with most digital intervention trials and may be subject to response bias, particularly at postpartum follow-up when demands on participants’ time are highest.

## 6 Public health and clinical implications

If effective, PROMATER could represent a meaningful contribution to the landscape of low-cost, scalable perinatal mental health resources in Brazil and other upper-middle-income settings with comparable structural constraints. Its digital format, brief duration, and evidence-based engagement strategies position it for potential integration into existing public health platforms, including those operating within the SUS framework. Beyond its direct clinical utility, the dismantling design of this trial will generate evidence on the active ingredients of compassion-based digital interventions – evidence that is currently sparse in the perinatal literature and that has the potential to inform the design of future programs across diverse settings and populations.

## 7 Ethics and dissemination

### 7.1 Ethics approval

The study protocol has been reviewed and approved by the Scientific Commission and the Research Ethics Committee of the Pontifical Catholic University of Rio Grande do Sul (PUCRS), under the Certificate of Presentation for Ethical Appreciation (CAAE: 90744125.8.0000.5336). All participants will be required to provide informed consent digitally before accessing the baseline assessment and being randomized into the study arms.

### 7.2 Safety and risk management

To ensure participant safety, suicidal ideation will be monitored at each assessment point using Item 10 of the EPDS, which assesses thoughts of self-harm on a four-point frequency scale (never, rarely, sometimes, always). Any score above 0 triggers an alert to the clinical research team. Response procedures are tiered by severity: participants scoring 1 (rarely) or 2 (sometimes) are contacted within 24 hours for priority assessment; participants scoring 3 (always) receive an immediate response and urgent referral to emergency psychiatric services. If significant distress is identified through other means, individualized guidance and referrals will be provided according to symptom severity and local service availability.

All safety procedures will adhere to predefined ethical protocols, prioritizing participant well-being, clinical support, and strict confidentiality. Participants requiring intensive psychiatric treatment will be advised to prioritize clinical care over study participation, and their data will be handled according to the Intention-to-Treat (ITT) principle.

### 7.3 Reporting standards

This protocol was developed and reported in accordance with the Standard Protocol Items: Recommendations for Interventional Trials (SPIRIT) guidelines (72). The completed SPIRIT 2025 checklist is provided as **Supplementary Material, Additional File 2**.

### 7.4 Dissemination policy

The results of this trial will be disseminated through peer-reviewed scientific journals, conference presentations, and reports in clinical trial registries (ReBEC/ICTRP). A plain language summary will be developed and shared with participants via email upon study completion. Additionally, findings may be communicated through social media and institutional platforms to increase public accessibility. No restrictions regarding publication are imposed by the funding agency.

## 8 Trial registration

The trial is registered at the Brazilian Registry of Clinical Trials (ReBEC) under registration number RBR-99tjfz5 (version 3.0, registered on December 11, 2025). The registration is publicly available at https://ensaiosclinicos.gov.br/rg/RBR-99tjfz5. The corresponding Universal Trial Number (UTN) is U1111-1332-0713. The study is also indexed in the WHO International Clinical Trials Registry Platform (ICTRP) and can be located using the ReBEC registration number (RBR-99tjfz5).
