# Supplementary material for: PROMATER: protocol for a randomized controlled trial of a digital self-guided intervention to promote perinatal mental health and reduce self-criticism
Source: Front Psychiatry. 2026 Sep 17;17:1943486. doi: 10.3389/fpsyt.2026.1943486 (PMC13646907; doi:10.3389/fpsyt.2026.1943486)
Supplement: Supplementary file 1 [file Table1.docx]

Supplementary Material

# 1 Additional File 1 - Intervention Components of the PROMATER Program

| **Module** | **Module Name** | **Main Topics** | **Key Interventions** | **Session Goal** |
| --- | --- | --- | --- | --- |
| **–** | **Welcome Session** | • Introduction to PROMATER and its objectives  • Overview of the 6-module structure  • Explanation of multimodal format (videos, infographics, audio practices, quizzes)  • Mention of 3-week duration and post-intervention assessment (T2) | • Welcome video  • Platform navigation orientation | Welcome participants and present the program's structure, objectives, and delivery format, fostering initial engagement and motivation. |
| **1** | **Expectations and Idealization of Motherhood** | • Emotional ambivalence: coexistence of love and difficulty in the perinatal period  • Myths of perfect motherhood reinforced by media and social networks  • Social comparison with peers, family, and influencers, and its impact on self-perception  • Expectations vs. lived reality of motherhood | • Video 1.1: Emotional ambivalence and myths of ideal motherhood; guided reflection on pre-pregnancy expectations  • Video 1.2: Social comparison; brief written exercise identifying self-comparative thoughts and questioning their validity  • One audio practice  • Infographic  • One quiz | Deconstruct idealizations of motherhood, normalize emotional ambivalence, and reduce self-demand arising from social comparisons. |
| **2** | **Understanding Self-Criticism** | • Definition of self-criticism as a harsh, rigid, and punitive internal stance  • Common maternal self-critical thoughts (e.g., “I am not a good mother”, “I am failing my baby”, “I should have done better”)  • Origins of the self-critical voice: developmental history, cultural, and social pressures  • Psychological impacts: association with depression, anxiety, shame, and guilt  • Physiological impacts: threat system activation, increased cortisol, HPA axis dysregulation, and reduced emotional responsiveness | • Video 2.1: Definition and examples of maternal self-criticism; origins of the critical voice  • Video 2.2: Psychological and physiological impacts of self-criticism; applied example of self-punishment after a parenting difficulty  • Video 2.3: Adaptation of the "Two Teachers Vignette" (Gilbert, 2009)  • Infographic  • One quiz | Increase awareness of the self-critical voice, understand its psychological and physiological impacts, and prepare participants for compassion-based coping strategies. |
| **3** | **Perinatal Mental Health** | • Symptoms of perinatal depression: persistent sadness, loss of interest, difficulty bonding with the baby, fatigue  • Symptoms of perinatal anxiety: excessive worry, constant alertness, physical tension  • Baby blues vs. postpartum depression: differentiation, duration, and clinical significance  • Risk factors, prevalence data, and early help-seeking as an act of self-care  • Perinatal loss | • Video 3.1: Overview of perinatal mental health; common emotional changes during pregnancy and postpartum; importance of early symptom recognition  • Video 3.2: Symptoms of perinatal depression and anxiety  • Video 3.3: Differentiation between baby blues and postpartum depression; when and how to seek professional help  • Video 3.4: Myths surrounding perinatal mental health  • Video 3.5: Perinatal loss  • Informative booklet on where and when to seek professional support  • Infographic  • One quiz | Provide psychoeducation on perinatal mood and anxiety disorders, promote early symptom recognition, and encourage professional help-seeking as an act of care rather than weakness. |
| **4** | **Social Support** | • Social support as a key protective factor for perinatal mental health  • Shift from community-based to dyadic parenting models and its impact on maternal isolation  • Types of support: practical (task-sharing), emotional (non-judgmental listening), and informational (professional guidance)  • Strategies for identifying, mobilizing, and expanding support networks  • Assertive communication: expressing needs clearly and respectfully | • Video 4.1: Importance of social support; types of support and their role in reducing perinatal stress; reflection on current support network  • Video 4.2: Practical strategies for strengthening the support network; assertive communication exercises (e.g., requesting help with childcare or household tasks); introduction to peer support groups  • Support network mapping exercise  • Assertive communication practice with applied examples  • Infographic  • One quiz | Recognize the importance of social support, identify and strengthen personal support networks, and develop assertive communication skills for requesting help. |
| **5** | **Self-Compassion** | • Concept of self-compassion: treating oneself with the same kindness offered to a loved one in distress  • Three components: self-kindness, common humanity, and mindfulness (Neff, 2003)  • Debunking myths: self-compassion vs. self-pity, self-indulgence, or irresponsibility  • Fears of compassion: “If I am gentle with myself, I will become lazy”  • Common humanity: normalizing shared maternal struggles  • Evidence-based benefits: reduction of anxiety, depression, and increased resilience | • Video 5.1: Definition of self-compassion; distinction from self-pity; self-compassion as a learnable resource  • Video 5.2: Fears of compassion; reframing self-compassion as motivation (not permissiveness); introduction to audio practices  • Video 5.3: Continuation of the Two Teachers Vignette  • Video 5.4: Benefits of self-compassion; self-compassionate phrase construction exercise  • Video 5.5: Common humanity reflection exercise  • One audio practice (soothing-rhythm breathing)  • One audio practice (self-compassion)  • One quiz | Introduce and practice self-compassion as a resource for coping with maternal distress, overcome fears related to self-kindness, and replace self-critical patterns with compassionate self-talk. |
| **6** | **Mindfulness and Mother–Infant Bonding** | • Part 1 – Mindfulness: concept of present-moment awareness; reduction of autopilot and ruminative thinking; stress and anxiety reduction  • Part 2 – Mother–infant bonding: demystification of immediate bonding; bonding as a gradual, daily process built through small gestures of care  • Practical applications in maternal daily routines (breathing, attention during feeding, skin-to-skin contact) | • Video 6.1: Mindfulness concept and benefits; practical applications in daily maternal routine; introduction to audio mindfulness practice; transition to mother–infant bonding  • Video 6.2: Mother–infant bonding as a gradual process; normalization of non-immediate bonding  • Video 6.3: Practical strategies to strengthen bonding (skin-to-skin contact, eye contact, talking, singing)  • Guided mindfulness audio practice  • Mindful presence exercise during baby care routines  • Infographic  • One quiz | Develop mindfulness skills to reduce stress and increase emotional presence, and strengthen the mother–infant bond through daily gestures of affective connection. |
| **–** | **Closing Session** | • Integrative synthesis of key learning points from all six modules  • Reinforcement of core messages: no single way to experience motherhood; resources can be revisited at any time  • Guidance on post-intervention assessment (T2) | • Closing video (Video 7): gratitude for participation; synthesis of all modules (emotional ambivalence, self-criticism, perinatal mental health, social support, self-compassion, mindfulness and bonding); encouragement to complete T2 questionnaires  • Integrative summary reviewing all modules and core messages | Consolidate program learnings, reinforce motivation to apply acquired resources in daily life, and guide participants toward the post-intervention assessment. |

# 2 Additional File 2 – SPIRIT Checklist

**SPIRIT 2025 checklist of items to address in a randomized trial protocol*** **PROMATER: Protocol for a randomized controlled trial of a digital self-guided intervention to promote perinatal mental health and reduce self-criticism**

| **Section / Topic** | **No** | **SPIRIT 2025 checklist item description** | **Reported on page no.** |
| --- | --- | --- | --- |
| **Administrative information** | | |  |
| Title and structured summary | 1a | Title stating the trial design, population, and interventions, with identification as a protocol | 1 |
|  | 1b | Structured summary of trial design and methods, including items from the World Health Organization Trial Registration Data Set | 1 |
| Protocol version | 2 | Version date and identifier | 13 |
| Roles and responsibilities | 3a | Names, affiliations, and roles of protocol contributors | 1 |
|  | 3b | Name and contact information for the trial sponsor | N/A |
|  | 3c | Role of trial sponsor and funders in design, conduct, analysis, and reporting of trial; including any authority over these activities | N/A |
|  | 3d | Composition, roles, and responsibilities of the coordinating site, steering committee, endpoint adjudication committee, data management team, and other individuals or groups overseeing the trial, if applicable | 9 |
| **Open science** | | |  |
| Trial registration | 4 | Name of trial registry, identifying number (with URL), and date of registration. If not yet registered, name of intended registry | 13 |
| Protocol and statistical analysis plan | 5 | Where the trial protocol and statistical analysis plan can be accessed | 10 |
| Data sharing | 6 | Where and how the individual de-identified participant data (including data dictionary), statistical code, and any other materials will be accessible | 19 |
| Funding and conflicts of interest | 7a | Sources of funding and other support (e.g., supply of drugs) | 14 |
|  | 7b | Financial and other conflicts of interest for principal investigators and steering committee members | 13 |
| Dissemination policy | 8 | Plans to communicate trial results to participants, healthcare professionals, the public, and other relevant groups (e.g., reporting in trial registry, plain language summary, publication) | 13 |
| **Introduction** | | |  |
| Background and rationale | 9a | Scientific background and rationale, including summary of relevant studies (published and unpublished) examining benefits and harms for each intervention | 2-4 |
|  | 9b | Explanation for choice of comparator | 4, 6 |
| Objectives | 10 | Specific objectives related to benefits and harms | 4-5 |
| **Methods: Patient and public involvement, trial design** | | |  |
| Patient and public involvement | 11 | Details of, or plans for, patient or public involvement in the design, conduct, and reporting of the trial | 8 |
| Trial design | 12 | Description of trial design including type of trial (e.g., parallel group, crossover), allocation ratio, and framework (e.g., superiority, equivalence, non-inferiority, exploratory) | 5 |
| **Methods: Participants, interventions, and outcomes** | | |  |
| Trial setting | 13 | Settings (e.g., community, hospital) and locations (e.g., countries, sites) where the trial will be conducted | 5 |
| Eligibility criteria | 14a | Eligibility criteria for participants | 5 |
|  | 14b | If applicable, eligibility criteria for sites and for individuals who will deliver the interventions (e.g., surgeons, physiotherapists) | N/A |
| Intervention and comparator | 15a | Intervention and comparator with sufficient details to allow replication including how, when, and by whom they will be administered. If relevant, where additional materials describing the intervention and comparator (e.g., intervention manual) can be accessed | 5-6 |
|  | 15b | Criteria for discontinuing or modifying allocated intervention/comparator for a trial participant (e.g., drug dose change in response to harms, participant request, or improving/worsening disease) | N/A |
|  | 15c | Strategies to improve adherence to intervention/comparator protocols, if applicable, and any procedures for monitoring adherence (e.g., drug tablet return, sessions attended) | 9 |
|  | 15d | Concomitant care that is permitted or prohibited during the trial | 11 |
| Outcomes | 16 | Primary and secondary outcomes, including the specific measurement variable (e.g., systolic blood pressure), analysis metric (e.g., change from baseline, final value, time to event), method of aggregation (e.g., median, proportion), and time point for each outcome | 6-8 |
| Harms | 17 | How harms are defined and will be assessed (e.g., systematically, non-systematically) | N/A |
| Participant timeline | 18 | Time schedule of enrollment, interventions (including any run-ins and washouts), assessments, and visits for participants. A schematic diagram is highly recommended (see Figure) | 8 |
| Sample size | 19 | How sample size was determined, including all assumptions supporting the sample size calculation | 8 |
| Recruitment | 20 | Strategies for achieving adequate participant enrollment to reach target sample size | 8 |
| **Methods: Assignment of interventions** | | |  |
| Randomization: |  |  |  |
| Sequence generation | 21a | Who will generate the random allocation sequence and the method used | 8 |
|  | 21b | Type of randomization (simple or restricted) and details of any factors for stratification. To reduce predictability of a random sequence, other details of any planned restriction (e.g., blocking) should be provided in a separate document that is unavailable to those who enroll participants or assign interventions | 8 |
| Allocation concealment  mechanism | 22 | Mechanism used to implement the random allocation sequence (e.g., central computer/telephone; sequentially numbered, opaque, sealed containers), describing any steps to conceal the sequence until interventions are assigned | 8 |
| Implementation | 23 | Whether the personnel who will enroll and those who will assign participants to the interventions will have access to the random allocation sequence | 9 |
| Blinding | 24a | Who will be blinded after assignment to interventions (e.g., participants, care providers, outcome assessors, data analysts) | 9 |
|  | 24b | If blinded, how blinding will be achieved and description of the similarity of interventions | 9 |
|  | 24c | If blinded, circumstances under which unblinding is permissible, and procedure for revealing a participant’s allocated intervention during the trial | 9 |
| **Methods: Data collection, management, and analysis** | | |  |
| Data collection methods | 25a | Plans for assessment and collection of trial data, including any related processes to promote data quality (e.g., duplicate measurements, training of assessors) and a description of trial instruments (e.g., questionnaires, laboratory tests) along with their reliability and validity, if known. Reference to where data collection forms can be accessed, if not in the protocol | 9 |
|  | 25b | Plans to promote participant retention and complete follow-up, including list of any outcome data to be collected for participants who discontinue or deviate from intervention protocols | 9-10 |
| Data management | 26 | Plans for data entry, coding, security, and storage, including any related processes to promote data quality (e.g., double data entry; range checks for data values). Reference to where details of data management procedures can be accessed, if not in the protocol | 9 |
| Statistical methods | 27a | Statistical methods used to compare groups for primary and secondary outcomes, including harms | 10 |
|  | 27b | Definition of who will be included in each analysis (e.g., all randomized participants), and in which group | 10 |
|  | 27c | How missing data will be handled in the analysis | 10 |
|  | 27d | Methods for any additional analyses (e.g., subgroup and sensitivity analyses) | 10 |
| **Methods: Monitoring** | | |  |
| Data monitoring committee | 28a | Composition of data monitoring committee (DMC); summary of its role and reporting structure; statement of whether it is independent from the sponsor and funder; conflicts of interest and reference to where further details about its charter can be found, if not in the protocol. Alternatively, an explanation of why a DMC is not needed | 10 |
|  | 28b | Explanation of any interim analyses and stopping guidelines, including who will have access to these interim results and make the final decision to terminate the trial | N/A |
| Trial monitoring | 29 | Frequency and procedures for monitoring trial conduct. If there is no monitoring, give explanation | 10 |
| **Ethics** | | |  |
| Research ethics approval | 30 | Plans for seeking research ethics committee/institutional review board approval | 12 |
| Protocol amendments | 31 | Plans for communicating important protocol modifications to relevant parties |  |
| Consent or assent | 32a | Who will obtain informed consent or assent from potential trial participants or authorized proxies, and how | 9 |
|  | 32b | Additional consent provisions for collection and use of participant data and biological specimens in ancillary studies, if applicable | N/A |
| Confidentiality | 33 | How personal information about potential and enrolled participants will be collected, shared, and maintained in order to protect confidentiality before, during, and after the trial | 9 |
| Ancillary and post-trial care | 34 | Provisions, if any, for ancillary and post-trial care, and for compensation to those who suffer harm from trial participation | N/A |

*We strongly recommend reading this checklist in conjunction with the SPIRIT 2025 Explanation and Elaboration and the SPIRIT 2025 Expanded Checklist for important clarifications on all the items. We also recommend reading relevant SPIRIT extensions. See [www.consort-spirit.org](http://www.consort-spirit.org)

Citation: Chan A-W, Boutron I, Hopewell S, Moher D, Schulz KF, et al. SPIRIT 2025 statement: updated guideline for protocols of randomised trials. BMJ 2025;389:e081477. <https://dx.doi.org/10.1136/bmj-2024-081477>

© 2025 Chan A-W et al. This is an Open Access article distributed under the terms of the Creative Commons Attribution License (<https://creativecommons.org/licenses/by/4.0/>), which permits unrestricted use, distribution, and reproduction in any medium, provided the original work is properly cited.

# 3 Additional File 3 – Obstetric and Postpartum Experience Questionnaire/Questionário sobre Experiência do Parto e Puerpério

# VERSÃO EM PORTUGUÊS

**Questionário sobre Experiência do Parto e Puerpério**

1. Idade do bebê (em meses): _______________
2. Data do parto: __ / __ / ____ _______________
3. Local do parto (cidade/estado): _______________
4. O período da gestação durou quantas semanas? _______________
5. Gestação gemelar?

☐ Não

☐ Sim → Se sim, quantos bebês? ___

6. Tipo de parto:

☐ Vaginal

☐ Cesárea

7. Índice de Apgar (1º e 5º minuto, se souber): ___ / ___ _______________

**Intervenções e condições clínicas**

8. Foram utilizadas intervenções médicas durante o parto? (Marque todas que se aplicam):

☐ Analgesia (ex: anestesia peridural ou raquidiana)

☐ Ocitocina sintética (para indução ou aceleração do parto)

☐ Fórceps

☐ Monitoramento fetal contínuo

☐ Ruptura artificial da bolsa amniótica

Outra: _______________

9. Peso ao nascer: ___ g	Altura ao nascer: ___ cm _______________
10. O bebê precisou ficar internado após o nascimento?

☐ Sim

☐ Não

Se sim, por quanto tempo? ___ Motivo: _______________

11. Você precisou ficar internada após o parto?

☐ Sim

☐ Não

Se sim, quanto tempo após o parto recebeu alta? _______________

**Experiências durante o parto e puerpério**

12. Você teve alguma complicação (emocional, psicológica ou física) durante o parto?

☐ Sim

☐ Não

Se sim, quais? _______________

13. Durante o parto ou atendimento na maternidade, você vivenciou alguma das situações abaixo?

(Pode marcar mais de uma opção)

☐ Foi submetida a algum procedimento médico sem explicação ou sem seu consentimento (ex: episiotomia, toques vaginais repetidos, cesárea não justificada, manobra de Kristeller)

☐ Foi tratada com palavras agressivas, humilhações ou repreensões por profissionais de saúde

☐ Sentiu-se ignorada ou desrespeitada ao tentar expressar dor, medo ou dúvidas

☐ Não teve o direito de estar acompanhada por uma pessoa de sua escolha

☐ Foi impedida de escolher a posição para o parto ou de se movimentar durante o trabalho de parto

☐ Foi separada do bebê sem explicações ou sem necessidade médica clara

☐ Não vivenciou nenhuma dessas situações

☐ Não tenho certeza

14. Você teve alguma complicação (emocional, psicológica ou física) após o parto?

☐ Sim

☐ Não

Se sim, quais? _______________

Aleitamento e uso de medicação

15. Como está o aleitamento atualmente?

☐ Apenas seio

☐ Seio + complemento

☐ Apenas complemento

16. Você teve dificuldades para amamentar?

☐ Sim

☐ Não

Se sim, quais? _______________

17. O bebê faz uso de alguma medicação atualmente?

☐ Sim

☐ Não

☐ Não sabe

Desde quando? _______________

Qual(is)? _______________

Temperamento do bebê

19. Com base na sua percepção, como você descreveria o temperamento do seu bebê?

(De 1 a 5, sendo 1 = muito difícil e 5 = muito fácil)

☐ 1	☐ 2	☐ 3	☐ 4	☐ 5

ENGLISH VERSION

**Obstetric and Postpartum Experience Questionnaire**

Baby's age (in months): _______________

Date of birth: __ / __ / ____ _______________

Place of birth (city/state): _______________

How many weeks did the pregnancy last? _______________

Multiple pregnancy (twins or more)?

☐ No

☐ Yes → If yes, how many babies? ___

Type of delivery:

☐ Vaginal

☐ Cesarean section

Apgar score (1st and 5th minute, if known): ___ / ___ _______________

**Interventions and clinical conditions**

Were any medical interventions used during labor and delivery? (Check all that apply):

☐ Analgesia (e.g., epidural or spinal anesthesia)

☐ Synthetic oxytocin (for induction or augmentation of labor)

☐ Forceps

☐ Continuous fetal monitoring

☐ Artificial rupture of membranes

Other: _______________

Birth weight: ___ g	Birth length: ___ cm _______________

Did the baby need to be hospitalized after birth?

☐ Yes

☐ No

If yes, for how long? ___ Reason: _______________

Did you need to remain hospitalized after delivery?

☐ Yes

☐ No

If yes, how long after delivery were you discharged? _______________

**Experiences during labor, delivery, and the postpartum period**

Did you experience any complications (emotional, psychological, or physical) during labor or delivery?

☐ Yes

☐ No

If yes, which ones? _______________

During labor, delivery, or your stay at the maternity ward, did you experience any of the following situations?

(You may select more than one option)

☐ You were subjected to a medical procedure without explanation or without your consent (e.g., episiotomy, repeated vaginal examinations, unjustified cesarean section, Kristeller maneuver)

☐ You were treated with aggressive language, humiliation, or reprimands by healthcare professionals

☐ You felt ignored or disrespected when trying to express pain, fear, or concerns

☐ You were denied the right to have a companion of your choice present

☐ You were prevented from choosing your delivery position or from moving during labor

☐ You were separated from your baby without explanation or without clear medical necessity

☐ None of the above situations occurred

☐ I am not sure

Did you experience any complications (emotional, psychological, or physical) after delivery?

☐ Yes

☐ No

If yes, which ones? _______________

**Breastfeeding and medication use**

How is breastfeeding going currently?

☐ Breastfeeding only

☐ Breastfeeding + formula supplementation

☐ Formula only

Did you experience any difficulties breastfeeding?

☐ Yes

☐ No

If yes, which ones? _______________

Is the baby currently taking any medication?

☐ Yes

☐ No

☐ Unknown

Since when? _______________

Which medication(s)? _______________

**Baby's temperament**

19. Based on your perception, how would you describe your baby's temperament?

(From 1 to 5, where 1 = very difficult and 5 = very easy)

☐ 1	☐ 2	☐ 3	☐ 4	☐ 5
